# Therapeutic potential of ERK5 targeting in triple negative breast cancer

**DOI:** 10.18632/oncotarget.2324

**Published:** 2014-11-04

**Authors:** María Jesús Ortiz-Ruiz, Stela Álvarez-Fernández, Tracy Parrott, Sara Zaknoen, Francis J. Burrows, Alberto Ocaña, Atanasio Pandiella, Azucena Esparís-Ogando

**Affiliations:** ^1^ Instituto de Biología Molecular y Celular del Cáncer. CSIC-IBSAL-Universidad de Salamanca. Spain; ^2^ Tragara Pharmaceuticals, Carlsbad, CA, USA; ^3^ Hospital Universitario de Albacete, and AECC Unit, Spain

**Keywords:** breast cancer, kinase inhibitor, ERK5

## Abstract

Triple negative breast cancers (TNBCs) account for 15% of all breast cancers, and represent one of the most aggressive forms of the disease, exhibiting short relapse-free survival. In contrast to other breast cancer subtypes, the absence of knowledge about the etiopathogenic alterations that cause TNBCs force the use of chemotherapeutics to treat these tumors. Because of this, efforts have been devoted with the aim of incorporating novel therapies into the clinical setting. Kinases play important roles in the pathophysiology of several tumors, including TNBC. Since expression of the MAP kinase ERK5 has been linked to patient outcome in breast cancer, we analyzed the potential value of its targeting in TNBC. ERK5 was frequently overexpressed and active in samples from patients with TNBC, as well as in explants from mice carrying genetically-defined TNBC tumors. Moreover, expression of ERK5 was linked to a worse prognosis in TNBC patients. Knockdown experiments demonstrated that ERK5 supported proliferation of TNBC cells. Pharmacological inhibition of ERK5 with TG02, a clinical stage inhibitor which targets ERK5 and other kinases, inhibited cell proliferation by blocking passage of cells through G_1_ and G_2_, and also triggered apoptosis in certain TNBC cell lines. TG02 had significant antitumor activity in a TNBC xenograft model in vivo, and also augmented the activity of chemotherapeutic agents commonly used to treat TNBC. Together, these data indicate that ERK5 targeting may represent a valid strategy against TNBC, and support the development of trials aimed at evaluating the clinical effectiveness of drugs that block this kinase.

## INTRODUCTION

Triple negative breast cancers (TNBCs) account for 15% of all breast cancers, and represent one of the most aggressive forms of the disease [1]. Despite important research efforts aimed at identifying molecular alterations in TNBC [2], the lack of known etiopathogenic alterations has prevented the development of targeted therapeutics in TNBC [3], and force the use of chemotherapeutics such as taxanes or vinorelbine for the management of TNBC [4, 5].

Kinases play important roles in the pathophysiology of several tumors, including TNBC [6]. ERK5 belongs to the MAP kinase family of signal transducers [7], and its deregulation has been linked to the pathophysiology of breast cancer [8]. A recent report has indicated that expression of ERK5 in breast tumors is linked to patient outcome, since patients with tumors with high levels of ERK5 have a poor prognosis as compared to those whose tumors do not overexpress ERK5 [9]. In vitro studies have shown that ERK5 is a critical mediator of growth factor signaling in hormone-receptor positive breast cancer cell lines, and expression of a dominant-negative form of ERK5 prevented growth factor-induced proliferation [8]. Moreover, in breast cancer cell lines which overexpress HER2, ERK5 is constitutively active and inhibition of ERK5 causes a decrease in proliferation of HER2+ cells [8, 9]. Furthermore, knockdown of ERK5 favored the action of drugs commonly used in the treatment of HER2+ breast cancer. Therefore, targeting of ERK5 may be of therapeutic relevance in those subtypes of breast cancer. These precedents raise the question of whether targeting ERK5 could be beneficial in the treatment of TNBC.

Here we have analyzed the expression of ERK5 in TNBC and the value of its targeting for the therapy of this disease. We show that ERK5 expression levels correlate with patient outcome in metastatic TNBC. The composite analyses of the biochemical, genetic and pharmacologic studies carried out in this work suggests that ERK5 targeting may be beneficial for the therapy of TNBC, and open the possibility of clinically testing drugs that act on ERK5 for the treatment of patients bearing this type of tumors.

## RESULTS

### ERK5 expression is linked to patient outcome in triple negative breast cancer

**Figure 1:**
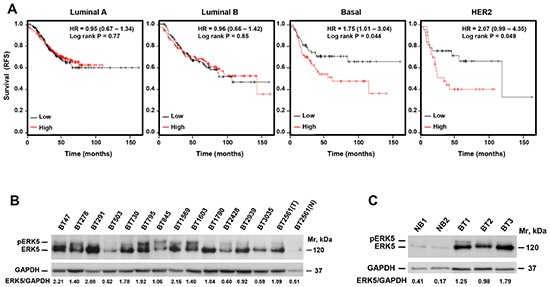
ERK5 expression is linked to patient outcome in TNBC **(A)** Kaplan-Meier analysis of the relationship between ERK5 expression and patient outcome in the different subgroups of breast cancer. **(B)** Western blotting analysis of the expression of ERK5 in tumors from patients with TNBC (in BT2561, N and T mean normal and tumoral breast tissue, respectively). **(C)** Expression of ERK5 in tumors from Brca1^−^/p53^−^ mice. NB: normal breast, BT: breast tumor. Expression of GAPDH was used as a loading control and the ratio between ERK5 and GAPDH expression was calculated in the tumor samples.

We evaluated the relationship between ERK5 expression and patient outcome in specific subsets of metastatic (lymph node positive) breast cancer. To this end, we used a publicly available database, which includes microarray expression data and clinical follow up from breast cancer patients [10]. Kaplan-Meier plots showed association between ERK5 expression and patient outcome in the basal type of breast cancer, which shares genomic and pathologic characteristics with TNBC, as well as in HER2+ tumors (Figure 1A). In contrast, these analyses failed to show any association between ERK5 expression and patient outcome in metastatic luminal A or metastatic luminal B.

The expression of ERK5 protein in tumor samples from patients with TNBC, analyzed by Western blot, showed that ERK5 was expressed in these samples, although at distinct levels (Figure 1B). Comparison of ERK5 levels of tumors samples to normal breast tissue (BT2561N) showed that ERK5 expression in TNBCs was higher than in the normal breast tissue. Interestingly, a number of tumor tissue samples contained a slower migrating form of ERK5, which corresponds to phosphorylated ERK5 (pERK5) [8]. We also explored ERK5 levels in tumors derived from mice with genomic and pathologic characteristics of TNBCs [11]. Tumors from these mice (BT1, BT2, and BT3) also displayed elevated levels of ERK5, as compared to non-tumoral (NB1, NB2) breast tissue (Figure 1C).

### ERK5 knockdown reduces the proliferation of triple negative breast cancer cells

**Figure 2:**
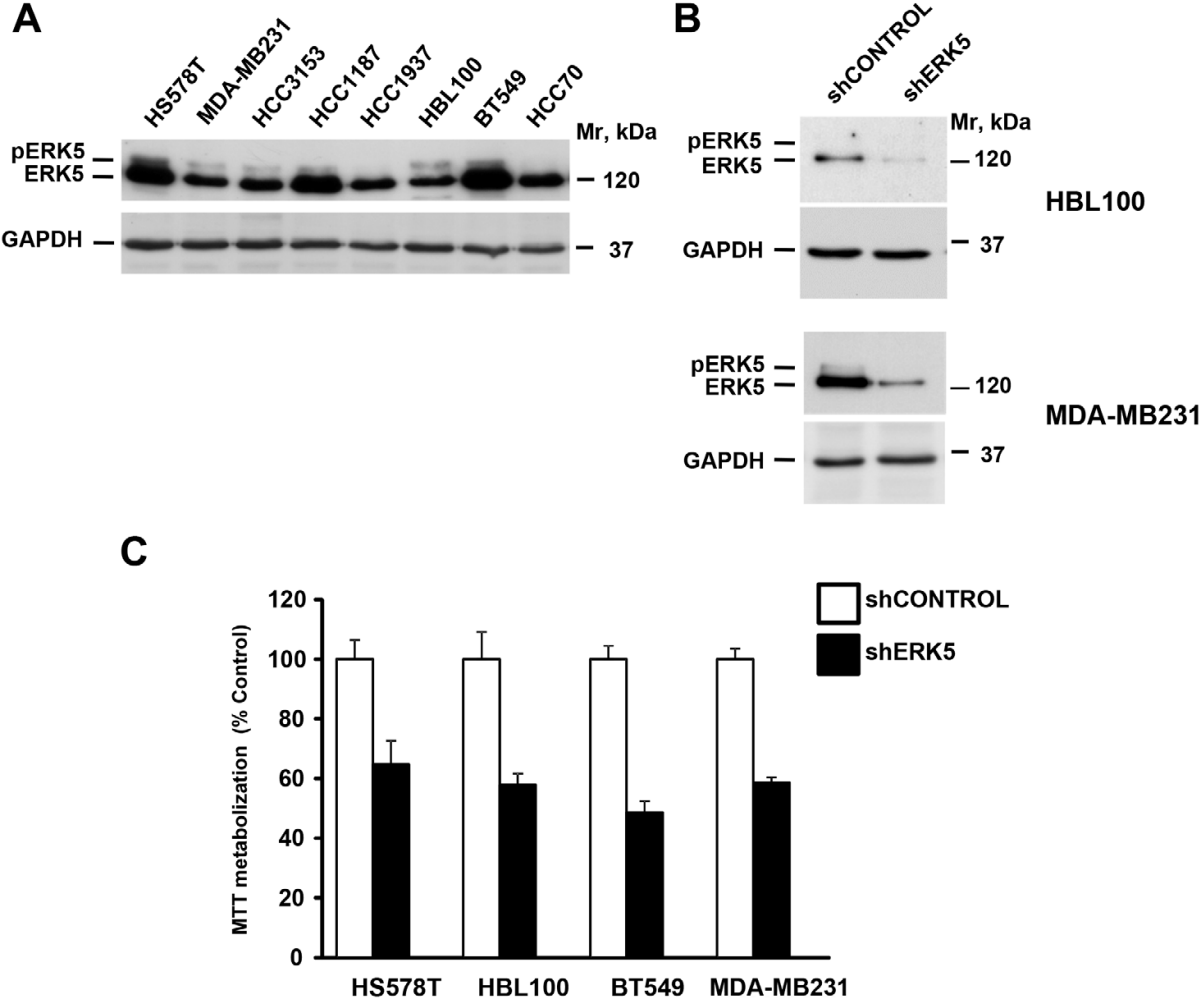
ERK5 controls proliferation of TNBC cell lines **(A)** Expression of ERK5 in TNBC cell lines. **(B)** Western blotting analyses of ERK5 expression in TNBC cells infected with a shControl or shERK5 lentiviral particles. Expression of GAPDH was used as a loading control. **(C)** Effect of ERK5 knockdown on the proliferation of TNBC cell lines. Cells were infected with lentiviral particles including a scrambled control sequence or a sequence targeting ERK5, and the proliferation measured by an MTT assay. Data are represented as mean ± SD of quadruplicates.

The elevated expression of ERK5 in TNBC, together with its prognostic relevance, led us to explore the value of targeting this kinase as a strategy against this type of tumors. For this purpose, we used human TNBC cell lines. All the cell lines tested expressed ERK5, and some of them contained phosphorylated forms of ERK5 (Figure 2A). We used knockdown of ERK5 to evaluate the impact of reducing its expression on the proliferation of TNBC on several of these cell lines. Western blotting demonstrated that infection with a shRNA that targets the mRNA for ERK5 reduced its protein levels (Figure 2B). Knockdown of ERK5 decreased their proliferation (Figure 2C). Knockdown experiments were also performed on HCC1187 cells. These cells proved to be extremely sensitive to ERK5 knockdown (Figure S1).

### Action of a clinical stage ERK5 inhibitor in triple negative breast cancer

The above data suggested that blocking ERK5 activity may have potential as a therapeutic strategy in TNBC. With the aim of translating these findings to the clinic, we used TG02, a pyrimidine-based macrocycle with ERK5 inhibitory properties [12], which may also target other kinases [13]. We selected this compound instead of other ERK5 inhibitors [14] because TG02 is the only clinical stage ERK5 inhibitor, currently in phase I studies (ClinicalTrials.gov Identifier: NCT01204164).

**Figure 3:**
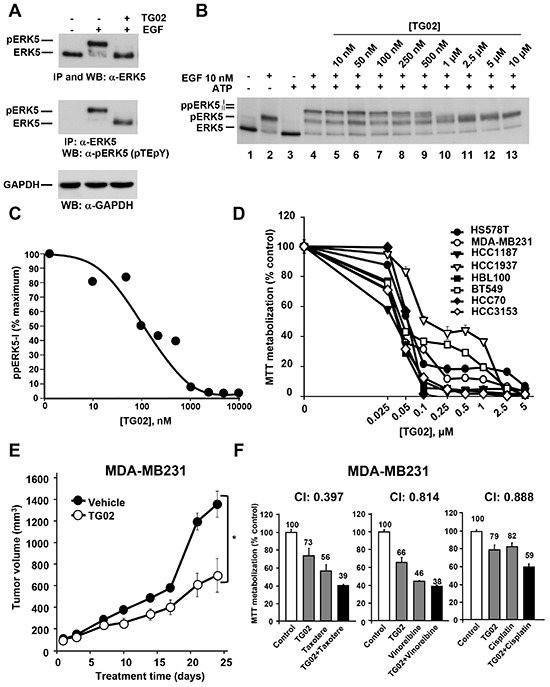
Effect of TG02 on ERK5 activity and on proliferation of TNBC cells **(A)**. MDA-MB231 cells were treated with EGF (10 nM, 15 minutes) in the absence or presence of TG02 (1 μM, 30 minutes). Cell lysates were immunoprecipitated with anti-ERK5 and the blots were probed with anti-ERK5 (top blot) or anti-pERK5 (middle blot). Expression of GAPDH was used as a loading control (bottom blot). **(B)** Effect of TG02 on ERK5 activity. ERK5 was immunoprecipitated from control or EGF-treated MDA-MB231 cells. Immunoprecipitated ERK5 was used for the in vitro kinase studies using the indicated concentrations of TG02. **(C)** Representation of the quantitative analysis of the amount of hyperphosphorylated ppERK5-I. **(D)** Dose-response effect of TG02 on different TNBC cell lines. Cells were incubated with increasing concentrations of TG02 for 72 hours and cell viability was analyzed by MTT metabolization. Data are represented as mean ± SD of quadruplicates, and results are shown as a percentage of control. **(E)** In vivo effect of TG02. Mice engrafted with MDA-MB231 cells were treated orally and daily with TG02 at a dose of 46.92 mg/kg. The graph represents the mean ± SEM of the tumor volumes. Differences in tumor growth between the treated group and the control (vehicle) were evaluated using the non-parametric Mann-Whitney U test. The asterisk indicates p<0.05. **(F)** MDA-MB231 cells were treated for 48 hours with TG02 (40 nM) and taxotere (4 nM), vinorelbine (3 nM) or cisplatin (7.5 μM) in monotherapy and in two-drug combinations. Cell viability was analyzed by MTT metabolization. The potency of the combination was analyzed with the CalcuSyn software and the combination index (CI) is shown at the top of each graph. Data are presented as mean ± SD of quadruplicates and results are shown as a percentage of control.

We evaluated the action of TG02 on ERK5 in intact TNBC cells. Under resting conditions, ERK5 migrates as a 120 kDa protein, and upon activation ERK5 undergoes a mobility shift easily detected by Western blotting [15]. That mobility shift is caused by phosphorylation of ERK5 at its TEY activation domain, as well as by phosphorylation of other residues at the C-terminal region of ERK5. Phosphorylations at the TEY microdomain are due to the ERK5 upstream activation kinase MEK5, while phosphorylations at the C-terminus depend on ERK5, and are expected to occur by autophosphorylation [16]. In MDA-MB231 cells treatment with EGF caused a mobility shift of ERK5 (Figure 3A, top panel), indicative of activation. Preincubation with TG02 inhibited EGF-induced ERK5 mobility shift, suggesting that TG02 was able to inhibit ERK5 in vivo. To demonstrate that this effect of TG02 was not due to inhibition of MEK5, we used an antibody which specifically recognizes ERK5 phosphorylated at its TEY microdomain [8]. Addition of EGF resulted in detection of pERK5 by this antibody (Figure 3A, middle panel). In cells treated with EGF, preincubation with TG02 caused a shift in the mobility of ERK5, which migrated faster. Yet ERK5 was recognized by the anti-pERK5 antibody, suggesting that the activity of MEK5 was preserved. Together, these data demonstrate that TG02 can inhibit ERK5 in live MDA-MB231 cells.

To verify that TG02 directly inhibited ERK5 activity we settled up a novel in vitro assay based on the kinase activity of ERK5 [12]. In this assay, ERK5 is used as both the enzyme and the substrate, because of its autophosphorylation properties. We used ERK5 isolated from non-stimulated as well as EGF-stimulated MDA-MB231 cells as a source of inactive or active kinase, respectively. When active ERK5 immunoprecipitated from MDA-MB231 cells treated with EGF was subjected to the in vitro assay, an additional shift in its mobility was observed, indicative of hyperphosphorylation (Figure 3B, lane 4). This supershift of ERK5 is due to autophosphorylation, and results in the appearance of two different bands which correspond to hyperphosphorylated ERK5 forms (ppERK5-I and pERK5-II). In the absence of EGF stimulation, immunoprecipitated ERK5 was unable to undergo any mobility shift (Figure 3B, lane 3). These data indicated that for these in vitro kinase assays ERK5 required previous in vivo activation. Incubation of active ERK5 with TG02 dose-dependently prevented the in vitro autophosphorylation of ERK5 (Figure 3B, lanes 5-13). Quantitative analyses of the fully hyperphosphorylated form (ppERK5-I) indicated that the in vitro inhibitory IC_50_ value of TG02 towards ERK5 was 100 nM (Figure 3C).

The action of TG02 was analyzed on eight TNBC cell lines. As shown in Figure 3D, growth and/or survival were inhibited by TG02 in all the TNBC cell lines. IC_50_ values were lower as incubation times increased (Figure S2A and S2B).

We next analysed the in vivo effect of TG02 on tumors derived by xenografting MDA-MB231. As shown in Figure 3E, treatment of mice injected with MDA-MB231 cells with TG02 delayed tumor growth.

As most antitumor treatments are based on combinations of drugs [17], we explored whether TG02 favoured the action of drugs used in the therapy of TNBC. Drug combinations were performed following the constant ratio protocol used in the Chou and Talalay algorithm [18]. Figure 3F shows the data for the best combinations as well as the combination indices obtained. TG02 synergized with conventional anti-TNBC treatments, as indicated by combination indices below 1. At the concentrations used for these combination experiments, the chemotherapeutic agents did not affect ERK5 status in live cells (Figure S2C). To evaluate whether these chemotherapeutics could directly affect ERK5 activity we performed in vitro kinase experiments. None of these treatments affected ERK5 activity (Figure S2D), neither at the concentrations used in the combination experiments (lanes 5-7), nor at much higher concentrations (Lanes 9-11). In contrast, TG02, used as a control, inhibited ERK5 activity. These results led us to conclude that the chemotherapeutic drugs used do not inhibit the activity of ERK5.

### TG02 delays cell cycle progression

**Figure 4:**
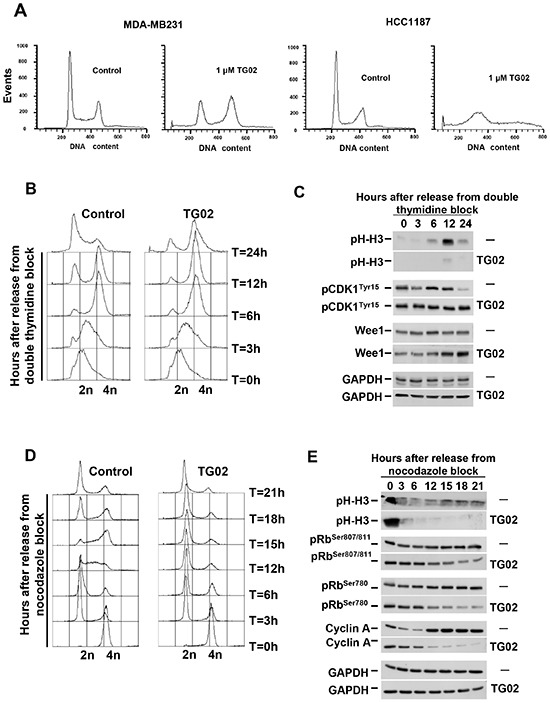
TG02 alters cell cycle progression **(A)** MDA-MB231 and HCC1187 cells were incubated with TG02 (1 μM for 24 hours), and the cell cycle profile was examined by flow cytometry. **(B-E)** MDA-MB231 cells were synchronized at late G1 by a double thymidine block **(B, C)** or in mitosis with nocodazole **(D, E)**, followed by release in the absence or presence of TG02 (100 nM). Cell cycle profiles were analyzed by FACS **(B, D)**. Protein extracts **(C, E)** were collected at different times after release, and proteins involved in cell cycle progression were analyzed by Western blot.

The decrease in MTT metabolization caused by TG02 could be due to a decrease in cell proliferation, augmented cell death, or both. To analyze this, we carried out propidium iodide (PI) staining of MDA-MB231 and HCC1187 cells treated with TG02. In MDA-MB231 cells, treatment with the drug caused accumulation of the cells in the G_2_/M phase (Figure 4A). The drug also increased the subG_0_ population, particularly in HCC1187, indicative of stimulation of cell death. These results indicated that the antitumor action of TG02 was complex and involved cell cycle and apoptotic mechanisms.

To analyse the effect of TG02 on the cell cycle we used synchronization agents that block cell cycle progression at different phases, followed by release from these blockers in the absence or presence of TG02. For these experiments, we used MDA-MB231 on which a clear effect of TG02 on the cell cycle was observed (Figure 4A).

Synchronization of MDA-MB231 cells at late G1 by a double thymidine block followed by release in the absence or presence of TG02 indicated that progression of cells along S phase was unaffected by treatment with the drug (Figure 4B). In fact, the PI histograms at 3 and 6 hours after release of control and TG02-treated cells were indistinguishable, and at 6 hours after release most of the cells had reached G_2_/M. In untreated cells, and 12-24 hours after release, a substantial amount of cells had progressed from mitosis into G_1_. In contrast, TG02-treated cells suffered a clear delay in their progression through the G_2_/M phase. Biochemically, phosphorylation of the mitotic marker histone H3 progressively increased in untreated cells (Figure 4C). In contrast, in TG02-treated cells phosphorylation of histone H3 was minimal. The lack of significant p-Histone H3 increases in cells treated with TG02 suggested that the drug impaired entry of cells into mitosis, and therefore could be impeding G2→M progression. Dephosphorylation of tyrosine 15 of CDK1 is required for entry into M phase [19]. Phosphorylation of this site is under the control of the kinase Wee1 and the phosphatase CDC25C. In control cells, pCDK1^Tyr15^ levels decreased after 12 hours. At this time, the amount of Wee1 was analogous to that present at other chase times, indicating that pCDK1^Tyr15^ dephosphorylation likely occurred by increased action of CDC25C. TG02 caused an accumulation of Wee1 (Figure 4C) and inhibited pCDK1^Tyr15^ dephosphorylation, which is indicative of CDK1/cyclin B complex inactivity.

To analyze whether TG02 also affects the transition through G1, MDA-MB231 cells were synchronized in mitosis with nocodazole, and then released in the absence or presence of TG02. Cell cycle profiles (Figure 4D) as well as phosphorylation of histone H3 (Figure 4E) confirmed that MDA-MB231 cells were arrested in mitosis after nocodazole blockade. TG02 did not substantially affect exit from mitosis, since 3 hours after release most of the cells had progressed into the G_1_ phase (Figure 4D). These results are in accordance with the data mentioned above indicating that TG02 acted on a step previous to the nocodazole-affected checkpoint. However, treatment with TG02 profoundly influenced the capability of MDA-MB231 cells to progress through the G_1_ phase into the S phase as demonstrated by comparison of the cell cycle profiles, particularly at 12 and 15 hours after release. Progression through the G1 phase requires the coordinate and sequential activation of several cyclin/CDK complexes [19]. Cyclins D1 and D3 in concert with CDK4/CDK6 act early in G1, and their activity is commonly detected by changes in Rb phosphorylation [20]. This facilitates the expression of other proteins involved in G1/S phase transition such as cyclin A, needed to activate CDK2 [21]. pRbSer^780^ and pRbSer^807/811^ levels decreased in cells treated with TG02 (Figure 4E). The gradual dephosphorylation of pRb in cells treated with TG02 could be responsible for the inhibition of cyclin A increase in expression, as compared with untreated MDA-MB231 cells.

### Effect of TG02 on apoptosis

**Figure 5:**
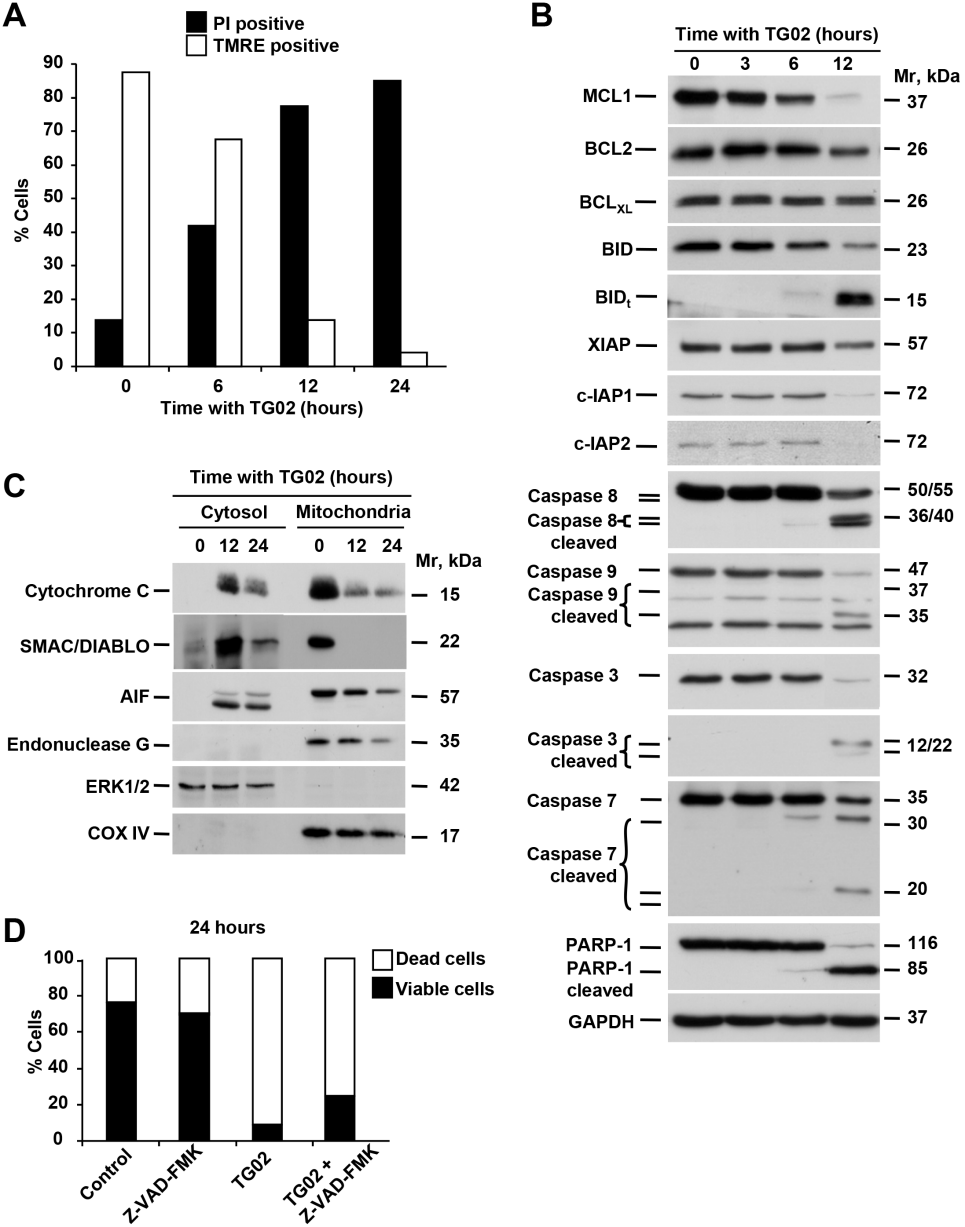
TG02 triggers apoptosis through caspase-dependent and independent pathways **(A)** HCC1187 cells were treated with TG02 (1 μM) for different times (0, 6, 12 and 24 hours). Loss of mitochondrial membrane potential and viability were analyzed by flow cytometry after staining with TMRE or propidium iodide (PI), respectively. **(B)** Expression of proteins involved in apoptotic mechanisms was analyzed by Western blot after treatment of HCC1187 cells with TG02 (1 μM) at the indicated times. GAPDH was used as loading control. **(C)** Release of proteins from the intermembrane space of mitochondria in HCC1187 cells treated for 12 and 24 hours with TG02 (1 μM) was analyzed by Western blot after subcellular fractionation. COX IV and ERK1/2 were analyzed as mitochondrial and cytosolic markers, respectively. **(D)** Role of caspases in TG02-induced cell death. Induction of cell death by TG02 was analyzed in HCC1187 cells by flow cytometry after 1 hour of pretreatment with the pan-caspase inhibitor Z-VAD-FMK (25 μM), followed by treatment with TG02 (1 μM) for 24 hours.

To analyse the mechanisms of apoptotic cell death triggered by TG02 we used HCC1187 cells. In this cell line TG02 had a more marked proapoptotic effect, assessed by propidium iodide staining and DNA laddering, as compared to MDA-MB231 (Figure 5A and Figure S3). Mitochondria play an important role in the induction of apoptosis through the release of apoptotic mediators that follows increased permeability of the outer mitochondrial membrane [22]. To investigate whether TG02 altered mitochondrial integrity we analyzed mitochondrial membrane potential (ΔΨm). Analysis by flow cytometry with TMRE revealed that TG02 provoked a progressive decrease in ΔΨm (Figure 5A).

Proteins of the BCL2 family act as major regulators of the mitochondrial outer membrane permeability [22]. TG02 decreased the levels of the antiapoptotic proteins MCL1 and BCL2 (Figure 5B). Moreover, treatment with TG02 provoked cleavage of the proapoptotic BCL2 family member BID, which may facilitate permeabilization of the outer mitochondrial membrane. To check if TG02 induced release of proapoptotic mediators from the mitochondrial intermembrane space, we carried out subcellular fractionation of HCC1187 cells. Cytochrome C along with the cytoplasmic protein Apaf-1 and pro-caspase 9 form the apoptosome, a macromolecular complex that causes the activation of effector caspase 3 [22]. As shown in Figure 5C, the amount of cytochrome C in mitochondria decreased after treatment with TG02, and an ensuing accumulation in the cytosol was observed. TG02 also decreased AIF and Endo G expression in the mitochondrial fraction. These two proteins are known to trigger apoptosis through caspase-independent mechanisms. In addition, TG02 treatment caused release of SMAC/DIABLO from the mitochondria. This protein acts as an inhibitor of the Inhibitor of Apoptosis (IAP) family of proteins [22], which were also down-regulated by TG02 in HCC1187 cells (Figure 5B).

The release of cytochrome C from the mitochondria to the cytosol suggested that caspases could contribute to TG02-induced cell death. Treatment with TG02 provoked fragmentation of caspases 8, 7, 9 and 3 (Figure 5B). In addition, cleavage of the caspase substrate PARP-1 was also observed in cells treated with TG02. The involvement of caspases in the death process induced by TG02 was further analyzed using the pancaspase inhibitor Z-VAD-FMK. HCC1187 cells were preincubated for 60 minutes with the caspase inhibitor Z-VAD-FMK and then treated with TG02. FACS analyses showed that Z-VAD-FMK partially inhibited TG02-induced death (Figure 5D), indicating that a proportion of the observed cell death was caspase-dependent.

## DISCUSSION

In this report, we show that ERK5 levels are frequently elevated in tumors from patients with TNBC. Furthermore, a relationship was found between high levels of ERK5 and worse prognosis in metastatic TNBC. In addition, we show that tumors from mice engineered to develop TNBC-like tumors also express elevated levels of ERK5 as compared to normal breast from the same mice. These data provided a springboard for further studies to determine the potential of ERK5 targeting in TNBC.

Experiments in which ERK5 was knocked down suggested that ERK5 targeting may be beneficial in TNBC, prompting us to evaluate the anti-TNBC activity of the clinical-stage inhibitor TG02. That drug decreased MTT metabolization of all of the TNBC lines tested at pharmacologically-achievable concentrations. Moreover, TG02 reduced tumor growth in vivo, and synergized with standard-of-care drugs used in the TNBC clinic, supporting the concept that TG02 may offer clinical benefit in this underserved cancer. Furthermore, since kinase addition appears to be a characteristic of tumors [23-25], agents such as TG02 which act on multiple kinases may be considered to expand the antitumoral therapeutic armamentarium.

The mechanisms behind cell death triggered by TG02 are likely to be multifactorial. TG02 provoked a delay in cell cycle progression and stimulated cell death. In MDA-MB231 cells, synchronization experiments indicated that treatment with the drug caused a delay in G_1_→S and G_2_→M transitions. ERK5 has been implicated in the regulation of G_1_→S [15, 26] and G_2_→M [14, 27] transitions, and this suggests that the action of TG02 on the cell cycle may rely, at least in part, on its ERK5-inhibitory properties. However, while ERK5 may influence cell cycle progression by regulating some cyclin/CDK complexes [26], a direct effect of TG02 on CDKs [13] cannot be excluded, and probably adds to the ERK5-dependent cell cycle regulatory properties.

TG02 activated a proapoptotic program in TNBC cells, as evidenced by increases in mitochondrial permeability which caused release of cytochrome C, SMAC/DIABLO, AIF and endonuclease G into the cytoplasm. Release of some of these mediators may be responsible for the cleavage and activation of caspases. The proapoptotic activity of TG02 is most probably driven by down regulation of survival proteins of the BCL2 family. TG02 inhibits CDK9 [13] and blockade of CDK9-dependent phosphorylation of RNA polymerase II reduces transcription rates [28], selectively depleting short-lived survival proteins including MCL-1, BCL2 and the IAP2 without altering expression of more stable relatives, such as BCL_XL_. In HCC1187 cells, TG02 treatment rapidly induced the activation of caspase-dependent cell death, an interpretation supported by the ability of the pan-caspase inhibitor Z-VAD-FMK to delay and partially restrict cell death. However, the partial inhibition of cell death using this compound indicated that caspase-independent mechanisms may also play a role in the action of TG02. In this respect, it is worth noting that AIF and endonuclease G may facilitate caspase-independent cell death when released from the mitochondria to the cytosolic and nuclear compartments [22].

In summary, our data show that ERK5 is frequently overexpressed in TNBC. Genetic knockdown studies demonstrated that ERK5 participated in the proliferation of TNBC cells, and treatment with the inhibitor TG02 had an antitumor effect in vitro and in vivo. These results, in addition to revealing the relevance of ERK5 in TNBC, open the door to the clinical evaluation of TG02 in the breast cancer clinic, especially in combination with standard- of-care chemotherapies used in the treatment of TNBC.

## METHODS

### Reagents and antibodies

Cell culture media, sera and penicillin-streptomycin were purchased from Invitrogen (Carlsbad, CA). Protein A-Sepharose was from GE Healthcare (Uppsala, Sweden). Taxotere was from Sigma Chemical (St Louis, MO), cisplatin was from Ferrer Farma (Barcelona, Spain), vinorelbine was from Pierre Fabre (Castres Cedex, France). Z-VAD-FMK and annexin V-FITC were from BD BioSciences (San Jose, CA). Other generic chemicals were purchased from Sigma Chemical (St. Louis, MO), Roche Biochemicals (Mannheim, Germany), or Merck (Darmstadt, Germany).

The antibodies utilized in the different Western blot analyses were: pCHK1^Ser296^, pCHK2^Tyr68^, pH2AX^Ser139^, caspase-7, caspase-9, COX IV and CDK9 (Cell Signalling Technology, Danvers, MA), GAPDH, PARP, pCDK1^Tyr15^, AIF, BCL2, MCL-1, Wee1 and ERK1/2 (Santa Cruz Biotechnology, Santa Cruz, CA); caspase-3, caspase-8, BCL_XL_, XIAP, cIAP1, cIAP2, cytochrome C, SMAC/DIABLO, cyclin A, pRb^Ser807/811^, pRbSer^780^ and Bid (BD Biosciences, San Jose, CA). Bid_t_ was provided by Dr. Huang (Walter and Eliza Hall Institute of Medical Research, Australia). Polyclonal antisera which recognized Endo G (Serotec, Oxford, UK) and phospho-histone H3 (Millipore, Bedford, MA) were used. The anti-ERK5 (C-terminal) and anti-ERK5-PRO1(R415) antibodies have been previously described [29]. Horseradish peroxidase–conjugated secondary antibodies were from Bio-Rad (Hercules, CA, USA).

### Patient samples and mice tumors

Analyses of the relationship between ERK5 and patient outcome was carried out using publicly available database, which allows estimation of the relevance of gene expression and clinical outcome in breast cancer (http://www.kmplot.com). TNBC samples from 14 patients were obtained from the Pathology Department of the University Hospital of Salamanca (Salamanca, Spain). Usage of these samples was approved by the Institutional Review Board Ethics Committee on Human Research of the Salamanca University Hospital.

Tumor samples from mice were obtained after sacrifice of the animals and immediately frozen in liquid Nitrogen. The Brca1/p53 conditional mice were generously provided by Dr. J. Jonkers (Netherlands Cancer Institute, Amsterdam). Mice were manipulated at the animal facility following legal and institutional guidelines.

For the detection of ERK5 expression by Western blot, the tumors were processed as previously described [9], and 1 mg of protein was immunoprecipitated with an anti-ERK5 antibody. As a control we used tissue derived from an area of normal breast tissue from patient BT2561 and normal breast tissue from mice.

### Cell culture, Western blotting and immunoprecipitation

The different TNBC cell lines used in this study were cultured in DMEM (Dulbecco's Modified Eagle Medium) containing high glucose (4.5 g/L), L-glutamine 4 mM and L-pyruvate 5 mM. The medium was supplemented with penicillin and streptomycin (100 U/ml and 100 μg/ml, respectively) and 5-10% FBS according to cell type. Cells were synchronized in mitosis with nocodazole for 14 hours or in late G_1_ with thymidine for 24 hours [30]. Production of lentivirus and lentiviral transduction assays, as well as analyses of proliferation, cell cycle, and apoptosis were performed as described [30]. Western blotting and immunoprecipitation were carried out as described [31].

### In vitro kinase assay

MDA-MB2321 cells treated with or without EGF (10 nM, 15 min) were collected and lysed in ice-cold lysis buffer; 1 mg of protein extract was immunoprecipitated with the anti-ERK5-PRO1(R415) antibody at 4°C for at least 2 h, and the immune complexes were recovered by a short centrifugation, followed by three washes with 1 ml of cold lysis buffer and two washes with 1 ml of kinase buffer (20 mM HEPES, pH 7.6; 20 mM MgCl_2_; 25 mM β-glycerophosphate). The immunoprecipitates were then incubated with different concentrations of TG02 (10-10 000 nM) for 1 hour at room temperature and after this time ATP and sodium orthovanadate were added to a final concentration of 100 μM and kinase reaction was performed at 30ºC for 30 min. Samples were analyzed by Western blotting with the anti-ERK5 (C-terminal) antibody and the ERK5 activity plotted in a graphic as a percent of control (maximum activity).

### In vivo studies

For the *in vivo* analyses, 9-10 week old *nu/nu* mice (Harlan Sprague Dawley, Inc. Indianapolis, IN) were subcutaneously inoculated into the right flank with 5 × 10^6^ MDA-MB231 cells and when tumors reached a size of 80-120 mm^3^, mice were randomized in two groups: Group 1 (control group that received the vehicle alone); and Group 2 (TG02 46.92 mg/kg orally every day). Tumor growth was evaluated twice a week and animals were euthanized when their tumors reached 1,500 mm^3^.

Statistical analyses were performed with the program SPSS-17.0 (SPSS Inc. Chicago, IL), and statistical significance was defined as p<0.05. All animal experiments were performed according to the protocols approved by the Charles River.

## SUPPLEMENTARY FIGURES


