# Supplementary material for: Therapeutic potential of ERK5 targeting in triple negative breast cancer
Source: Oncotarget. 2014 Nov 4;5(22):11308–18. doi: 10.18632/oncotarget.2324 (PMC4294347; doi:10.18632/oncotarget.2324)
Supplement: Supplementary file 1 [file oncotarget-05-11308-s001.pdf]

SUPPLEMENTARY FIGURES

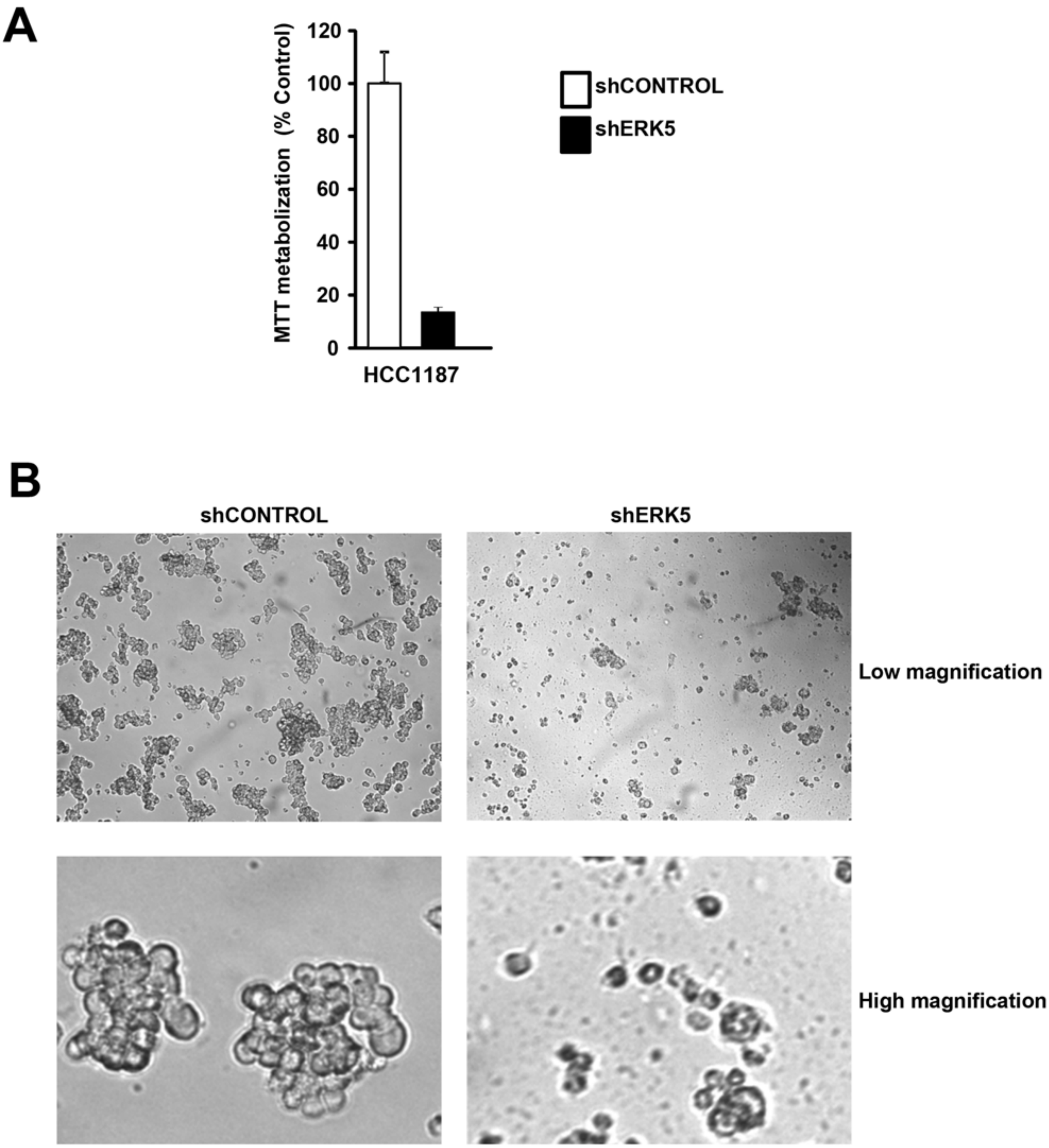

Supplementary Figure S1: (A) Effect of ERK5 knock down on the proliferation of HCC1187 cells. Cells were infected with retroviral particles including a scrambled control sequence or a sequence targeting ERK5, and the proliferation measured by an MTT assay. Data are represented as mean ± SD of quadruplicates. **(B)** Representative micrographs taken at low and high magnification of cultures of HCC1187 cells infected with retroviral particles including a scrambled control sequence or a sequence targeting ERK5.

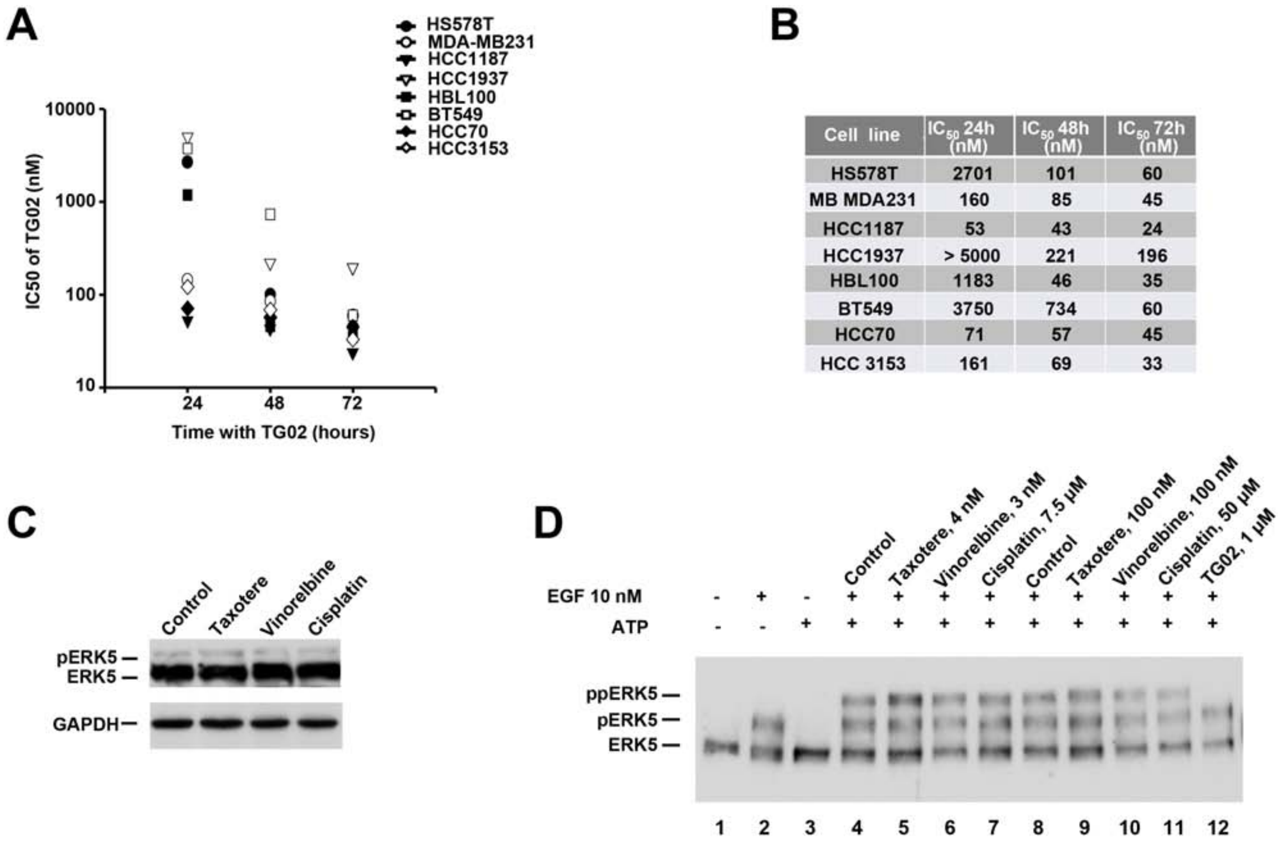

Cell line	IC_{50} 24h (nM)	IC_{50} 48h (nM)	IC_{50} 72h (nM)
HS578T	2701	101	60
MB MDA231	160	85	45
HCC1187	53	43	24
HCC1937	> 5000	221	196
HBL100	1183	46	35
BT549	3750	734	60
HCC70	71	57	45
HCC 3153	161	69	33

Supplementary Figure S2: IC_{50} values of the effect of TG02 on the proliferation of TNBC cells. (A) Shows a graphic with the representation of the IC_{50} values for the different cell lines at the distinct times (24, 48 and 72 hours) at which these values were calculated; and **(B)** details the values. **(C)** Effect of different chemotherapeutics on ERK5 activation status measured by Western blotting. Taxotere, vinorelbine or cisplatin were added for 48 hours at the concentrations indicated in Figure 3F. ERK5 and pERK5 were evaluated in 100 µg of cell lysates using the anti-ERK5 antibody. **(D)** Effect of taxotere, vinorelbine or cisplatin on ERK5 activity. ERK5 was immunoprecipitated from control or EGF-treated MDA-MB231 cells. Immunoprecipitated ERK5 was incubated with the indicated concentrations of the drugs, and in vitro kinase reactions performed as described under the methods section. TG02 was used as a control which inhibited ERK5 activity.

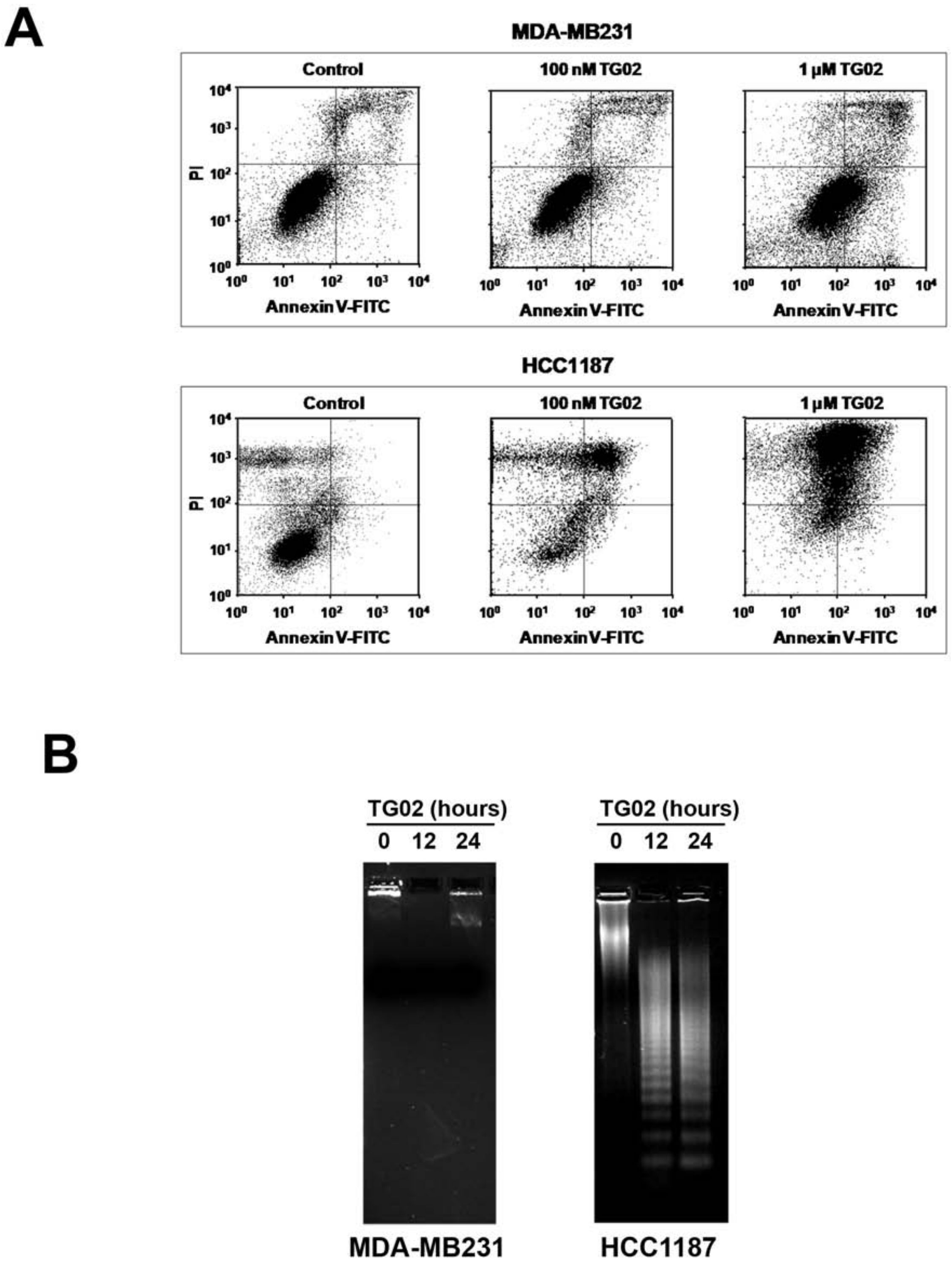

Supplementary Figure S3: TG02 induces apoptosis in TNBC cells. (A) MDA-MB231 and HCC1187 cells were incubated with TG02 at doses of 100 nM and 1 μM for 24 hours, and induction of apoptosis was examined by flow cytometry. **(B)** To detect DNA laddering, the cells were seeded and incubated for 0, 12 and 24 hours with 1 μM TG02. DNA was isolated and analyzed by agarose gel electrophoresis.
